# Novel Monoclonal Antibody Specific toward Amyloid-β Binds to a Unique Epitope within the N-Terminal Region

**DOI:** 10.3390/antib13030068

**Published:** 2024-08-09

**Authors:** Giavanna Paterno, Brenda D. Moore, Brach M. Bell, Kimberly-Marie M. Gorion, Yong Ran, Stefan Prokop, Todd E. Golde, Benoit I. Giasson

**Affiliations:** 1 Department of Neuroscience, College of Medicine, University of Florida, Gainesville, FL 32610, USA; giavanna@ufl.edu (G.P.); brachbell@ufl.edu (B.M.B.); kimgorion@ufl.edu (K.-M.M.G.); 2 Center for Translational Research in Neurodegenerative Disease, College of Medicine, University of Florida, Gainesville, FL 32610, USA; sprokop@ufl.edu; 3 Department of Pharmacology and Chemical Biology, Emory University School of Medicine, Atlanta, GA 30322, USA; brenda.dawn.moore@emory.edu (B.D.M.); yong.ran@emory.edu (Y.R.); todd.golde@emory.edu (T.E.G.); 4 Center for Neurodegenerative Disease, Emory University School of Medicine, Atlanta, GA 30322, USA; 5 McKnight Brain Institute, College of Medicine, University of Florida, Gainesville, FL 32610, USA; 6 Department of Pathology, College of Medicine, University of Florida, Gainesville, FL 32610, USA

**Keywords:** Amyloid-β, Alzheimer’s disease, antibody, pathology, plaques

## Abstract

Amyloid-β (Aβ) deposition throughout the neuroaxis is a classical hallmark of several neurodegenerative diseases, most notably Alzheimer’s disease (AD). Aβ peptides of varied length and diverse structural conformations are deposited within the parenchyma and vasculature in the brains of individuals with AD. Neuropathologically, Aβ pathology can be assessed using antibodies to label and characterize their features, which in turn leads to a more extensive understanding of the pathological process. In the present study, we generated a novel monoclonal antibody, which we found to be specific for the N-terminal region of Aβ. This antibody reacted to amyloid precursor protein expressed in cultured cells and labels Aβ plaques and cerebral amyloid angiopathy in brain tissue from a mouse model of amyloidosis as well as post-mortem brain tissue from patients diagnosed with AD. This highly specific novel antibody will serve as a unique tool for future studies investigating Aβ deposition in novel mouse models and cross-sectional studies using post-mortem human tissue.

## 1. Introduction

Alzheimer’s disease (AD) is a neurodegenerative disorder marked clinically by progressive and irreversible cognitive decline and memory loss [1]. AD is recognized as a proteinopathy in which aggregated tau and amyloid-β (Aβ) proteins are aberrantly deposited throughout regions of the central nervous system [2,3,4]. The presence of both pathologies with appreciable abundance and specific neuroanatomical distribution is required for definitive diagnosis, making these lesions the quintessential neuropathological hallmarks of AD [5].

Aggregated Aβ is found to accumulate within the parenchyma as extracellular plaques and within the vasculature, termed cerebral amyloid angiopathy (CAA) [6,7]. Initial studies on the biochemical isolation of Aβ from the brains of individuals with AD led to the discovery of the primary amino acid sequence of the peptide comprising plaques and CAA [8,9,10], and subsequent studies determined Aβ was derived from the amyloid precursor protein (APP) via proteolytic cleavage [11]. Aβ is particularly prone to aggregate due to its high hydrophobic character [12,13]. Ultrastructurally, Aβ plaques are composed of fibrils and are typified by an amyloid structure defined by a cross-β pleated sheet conformation [14,15,16,17]. Further ultrastructural analyses using cryo-electron microscopy have determined the structure of Aβ fibrils from AD brain tissue, revealing polymorphic folds of the ordered cores of Aβ which differed between filaments isolated from the cortex compared to the leptomeninges [18,19].

Early histochemical studies found that Aβ plaques in AD brain could be distinguished using amyloid-specific dyes such as Thioflavin-S and Congo Red [20,21], and further sensitive immunocytochemical methods were utilized, which were able to reveal a full spectrum of Aβ deposits [22]. Immunochemical techniques have been used to investigate the biochemical and morphological characteristics of Aβ deposits in the brains of patients with AD, Aβ co-pathology in Lewy body diseases, and Aβ immunoreactivity in the brains of neurologically normal individuals [23,24,25,26,27,28,29,30,31,32]. These plaque types have been characterized according to morphology, associations with genetic factors, neuropathological progression, and, importantly, correlation with AD clinicopathological phenotypes [2,7,33,34,35,36,37].

The exact role Aβ has in contributing to AD pathogenesis has spurred multiple hypotheses, including the amyloid cascade hypothesis, which posits that Aβ plaque accumulation acts as a nidus for successive pathological events such as tau aggregation and neurodegeneration [38,39]. Indeed, biomarker studies have shown that Aβ is one of the first pathologies to manifest years prior to symptom onset [40,41,42,43], making it a paramount area of investigation for disease-modifying therapeutics, in particular, monoclonal antibody treatment. Monoclonal antibodies specific for Aβ have considerable potential as therapies to relieve Aβ plaque burden with the goal that AD symptoms will be attenuated, but the pathological species to target and which will offer the most relief of clinical symptoms without increasing unwanted side effects remains under investigation. Antibodies used in clinical trials have been assessed for their abilities to engage distinct Aβ species [44] but also have shown diverse clinical outcomes [45,46,47,48,49,50,51,52]. Therefore, novel monoclonal antibodies that bind to epitopes on Aβ distinct from that of previously described antibodies may prove clinically useful.

In the present study, we generated and characterized a novel mouse monoclonal antibody specific for Aβ referred to as 3C11. Characterization was carried out using enzyme-linked immunosorbent assay (ELISA), western blotting, and immunohistochemistry to determine the epitope and investigate reactivity for AD neuropathological hallmarks. The monoclonal antibody 3C11 will serve as a unique tool for neuropathological studies investigating APP and Aβ deposition in murine models and AD human brain tissue.

## 2. Materials and Methods

### 2.1. Generation of Novel Anti-Aβ Mouse Monoclonal Antibody

The cDNA sequence of residues 21–140 of wild-type human α-synuclein with an added ATG start codon at the 5′ end followed by codons for residues 1–42 of human Aβ with a stop codon (hereafter referred to as 21–140 αS/Aβ 1–42) was synthesized as a service provided by GenScript (Piscataway, NJ, USA), cloned into the bacterial expression vector pET16b, and expressed in *Escherichia coli* (*E.coli*) BL21 (DE3)-RIL (Agilent Technologies, Santa Clara, CA) as previously described [53,54]. The chimeric protein (21–140 αS/Aβ 1–42) was purified using a HiTrap Q HP anion exchange chromatography column followed by size exclusion chromatography. Protein concentration was determined by bicinchoninic acid (BCA) assay using bovine serum albumin (BSA) as the standard (Pierce, Rockford, IL, USA). 21–140 αS/Aβ 1–42 was used for immunization of female BALB/c mice (Jackson Laboratory, Bar Harbor, ME, USA) as previously described [53,54]. All procedures were performed according to the NIH Guide for the Care and Use of Experimental Animals and were approved by the University of Florida Institutional Animal Care and Use Committee. 21–140 αS/Aβ 1–42 was emulsified in Freunds complete adjuvant (Sigma Aldrich, St. Louis, MO, USA) and injected subcutaneously. Three weeks later, the immunization peptide was emulsified in Freunds incomplete adjuvant (Sigma Aldrich, St. Louis, MO, USA) and injected intraperitoneally, followed by a booster intraperitoneal injection after an additional three weeks. Three days later, mice were euthanized by CO_2,_ spleens were harvested, and white blood cells were fused with mouse myeloma cells (Sp2/O-Ag14; ATCC, Manassas, VA, USA). Hybridoma clones were selected using HAT supplement (Sigma Aldrich, St. Louis, MO, USA), and the surviving clones were initially screened for reactivity by enzyme-linked immunosorbent assay (ELISA).

### 2.2. Antibodies

6E10 is an IgG_1_ mouse monoclonal antibody specific for human APP with an epitope reported within Aβ 1–16 [24]. Ab5 is an IgG_2b_ mouse monoclonal antibody raised against fibrillar Aβ 42; it has an epitope within Aβ 1–16 [55]. GA1R is an IgG_1_ mouse monoclonal antibody specific for GAPDH (Thermo Scientific, Waltham, MA, USA). 3C11 is an IgG_1_ mouse monoclonal antibody described herein. Antibody isotype of 3C11 was determined by ELISA using Mouse Monoclonal Antibody Isotyping Reagents (Sigma Aldrich, St. Louis, MO, USA). For western blotting and ELISA, antibodies were used at 1:1000. For immunohistochemical staining, antibodies were used at 1:5000.

### 2.3. Tissue Processing and Immunohistochemistry

Human brain tissue was obtained from the University of Florida Neuromedicine Human Brain and Tissue Bank (HBTB) with approval by the institutional review board. Human brain tissue was fixed using formalin, paraffin-embedded, and sectioned. Mice were euthanized with CO_2_ and perfused with a heparin/phosphate buffered saline (PBS; 11.9 mM phosphates, pH 7.4, 137 mM NaCl, 2.7 mM KCl) solution. Mouse tissue was harvested and fixed using 70% EtOH/150 mM NaCl, embedded in paraffin, and sectioned. Human and mouse brain tissue sections were deparaffinized in xylenes and rehydrated in a descending series of ethanol solutions (100%, 100%, 90%, 70%) followed by water. For human brain tissue, sections were then incubated in 70% formic acid for 20 min followed by rinsing in water (this step was omitted for mouse tissue). Heat-induced epitope retrieval was then performed in a steam bath for 1 h in water, followed by a water rinse. Endogenous peroxidases were quenched by incubating tissue sections in 1.5% hydrogen peroxide and 0.005% Triton X-100 in PBS for 15–20 min follwed by water rinse. Slides were incubated in 0.1 M Tris, pH 7.6 and then blocked with 2% fetal bovine serum (FBS)/0.1 M Tris, pH 7.6. Primary antibodies were diluted in blocking solution and applied to slides overnight at 4 °C. The following day, slides were washed in 0.1 M Tris, pH 7.6 and blocked in 2% FBS/0.1 M Tris, pH 7.6. Horse anti-mouse ImmPRESS polymer IgG secondary antibody (Vector Laboratories, Newark, CA, USA) (1:10) and biotinylated goat anti-mouse IgG secondary antibody (Vector Laboratories, Newark, CA, USA) (1:3000) were diluted in blocking solution and applied to slides for 1 h at room temperature (RT). Slides were washed with 0.1 M Tris, pH 7.6, and blocked with 2% FBS/0.1 M Tris, pH 7.6. An avidin–biotin complex (ABC) solution (Vectastain ABC kit; Vector Laboratories, Newark, CA, USA) was diluted 1:3000 and applied to slides for 1 h at RT. Slides were washed in 0.1 M Tris, pH 7.6, and tissue sections were developed using the chromogen 3,3′-diaminobenzidine (DAB kit, KPL, Gaithersburg, MD, USA). The reaction was stopped by incubating sections in water, and tissue was then counterstained using hematoxylin (Sigma Aldrich, St. Louis, MO, USA). Sections were washed in tap water, dehydrated in alcohols (70%, 90%, 100%, 100%), followed by xylenes, and coverslipped using cytoseal. Stained sections were digitally scanned at 40x magnification using an Aperio Scan Scope AT2 instrument, and images were captured using the ImageScope software (Version v12.4.3.5008; Aperio Technologies Inc., Vista, CA, USA).

### 2.4. Quantification of Aβ Plaques in Human Brain Tissue

Frontal cortex tissue from 12 AD cases (see Table 3 below) was used for quantification of Aβ plaques immunostained with antibodies 3C11 and Ab5. Three fields of view were captured at 10× magnification using ImageScope software (Version v12.4.3.5008; Aperio Technologies Inc., Vista, CA, USA), and dense Aβ plaques were manually counted by two investigators. For each case per antibody, the Aβ plaque counts were averaged, and a ratio was determined (3C11/Ab5) to provide a measure of 3C11 immunoreactivity compared to that of Ab5.

### 2.5. Enzyme-Linked Immunosorbent Assay (ELISA)

Aβ peptides were synthesized as a service provided by GenScript (Piscataway, NJ, USA) or AnaSpec (Fremont, CA, USA). Peptides were solubilized in either dimethyl sulfoxide at 5 or 1 mg/mL and stored at −80 °C or in 1,1,1,3,3,3-hexafluoroisopropanol at 1 mg/mL and then dried, aliquoted, and stored at −20 °C. Next, dried peptides were reconstituted in 50 mM NaOH and then PBS to 1 mg/mL. Peptides were diluted to 1 µg/mL in PBS, and 100 µL was adsorbed onto each well of Immulon 4HBX plates (Method 1) or MaxiSorp plates (Method 2) (ThermoFisher Scientific, Waltham, MA, USA) and incubated overnight at 4 °C. The following day, plates were washed with PBS and blocked with 1% Block ACE (Bio-Rad, Hercules, CA, USA) diluted in H_2_O overnight at 4 °C (Method 1) or 5% FBS/PBS for approximately 1 h at RT (Method 2). Primary antibodies were diluted in buffer EC (5 mM NaH_2_PO_4_, 20 mM Na_2_HPO_4_, pH 7.0, 400 mM NaCl, 2.55 mM EDTA, 1% BSA, 0.05% CHAPS) (Method 1) or 5% FBS/PBS (Method 2), applied to wells, and incubated at RT followed by PBS washes. Plates were incubated with goat anti-mouse secondary antibody conjugated to horse-radish peroxidase (HRP) (Jackson Immuno Research Labs, West Grove, PA, USA) in Buffer C (3 mM NaH_2_PO_4_, 20 mM Na_2_HPO_4_, pH 7.0, 400 mM NaCl, 1.98 mM EDTA, 1% BSA) (Method 1) or 5% FBS/PBS (Method 2) at RT for 1 h. Plates were washed with PBS-T (0.1% Tween-20/PBS) followed by PBS (Method 1) or PBS only (Method 2). 3,3′,5,5′-tetramethylbenzidine was used as a chromogenic substrate for HRP, which was added to the wells and allowed to develop until a color change was observed. Reactions were stopped using 1 N HCl or 5.7% O-phosphoric acid, and optical density was measured at 450 nm using a plate reader.

### 2.6. Calcium Phosphate Transfection and Cell Harvest

HEK293T cells were cultured in Dulbecco’s Modified Eagle Medium with L-Glutamine and high glucose (4.5 g/L) supplemented with 10% FBS and antibiotics (100 U/mL penicillin and 100 µg/mL streptomycin) at 37 °C and 5% CO_2_. Cells were transfected using calcium phosphate precipitation as described previously [56] with some modifications. 3 µg of DNA was added to 37.5 µL of 0.25 M CaCl_2_ and mixed. 37.5 µL of 2X BES buffer (50 mM *N*,*N*-bis(2-hydroxyethyl)-2-aminoethanesulfonic acid, 280 mM NaCl, 1.5 mM Na_2_HPO_4_, pH 6.96) was added step-wise 1/5th at a time to the DNA/CaCl_2_ mixture with vortexing after each addition. The transfection solution was incubated at RT for 15–20 min before adding to cells drop-wise. Approximately 16 h after transfection, the medium was replaced with Dulbecco’s Modified Eagle Medium with 3% FBS supplemented with antibiotics. Cells were harvested 48 h thereafter. For total cell harvest, samples were collected in Triton lysis buffer (25 mM Tris-HCl, pH 7.5, 150 mM NaCl, 1 mM EDTA, 1% Triton X-100, 20 mM NaF) supplemented with protease inhibitors (1 mM phenylmethylsulfonyl fluoride and 1 µg/mL each of pepstatin, leupeptin, *N*-tosyl-L-phenylalanyl chloromethyl ketone, *N*-tosyl-lysine chloromethyl ketone and soybean trypsin inhibitor). 5X SDS-sample buffer was added to a final concentration of 1X (10 mM Tris, pH 6.8, 1 mM EDTA, 40 mM DTT, 0.005% Bromophenol Blue, 0.0025% Pyronin Yellow, 1% SDS, 10% Sucrose) and samples were heated at 95 °C for 10 min and stored at −80 °C until further use.

### 2.7. Western Blotting

Equal volumes of HEK293T cell lysates were loaded onto 8% SDS-polyacrylamide gels and resolved by SDS-PAGE [57]. Proteins were electrophoretically transferred onto nitrocellulose membranes and further blocked with 5% nonfat dry milk in Tris-buffered saline (TBS; 50 mM Tris, pH 7.5, 150 mM NaCl) for 1 h at RT. Primary antibodies were diluted in a blocking solution, and membranes were incubated in primary antibodies overnight at 4 °C. The following day, membranes were washed three times in TBS and incubated in anti-mouse secondary antibody conjugated to HRP (Jackson ImmunoResearch, West Grove, PA, USA) for 1 h at RT. Membranes were then washed three times in TBS and developed using Western Lightning Plus ECL reagents (PerkinElmer, Waltham, MA, USA) and imaged using chemiluminescence.

## 3. Results 

### 3.1. Epitope Characterization of Novel Monoclonal Antibody 3C11

**Figure 1:**
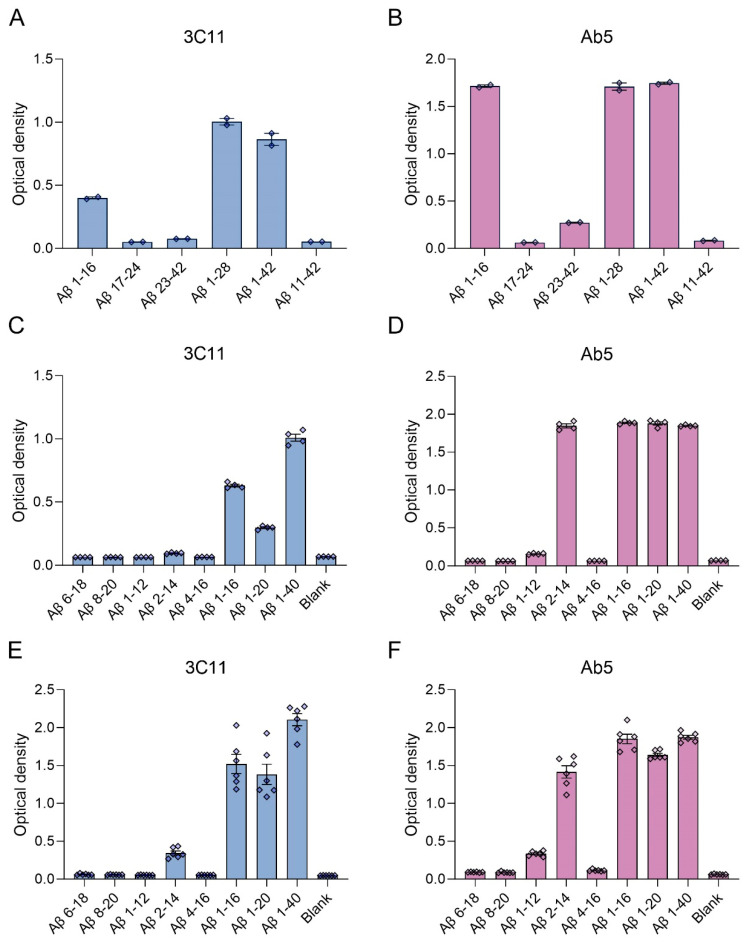
ELISA characterization of the novel mouse monoclonal antibody 3C11 using synthetic Aβ peptides. ELISA using method 1 (**A**–**D**) or 2 (**E**,**F**) was performed as described in Section 2. Plates were coated with synthetic Aβ peptides shown in Table 1 or uncoated (Blank) followed by incubation with 3C11 (**A**,**C**,**E**) or Ab5 (**B**,**D**,**F**). 3C11 immunoreactivity was compared to antibody Ab5. Data are shown as mean +/− SEM.

**Figure 2:**
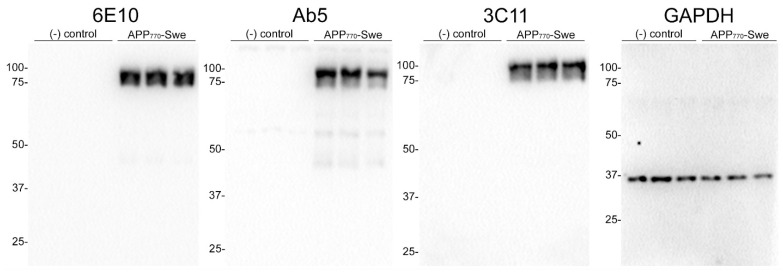
Immunoblot analysis demonstrating specificity of 3C11 monoclonal antibody. HEK293T cells were transfected in triplicate using plasmids expressing Amyloid Precursor Protein (APP) isoform 770 with familial AD Swedish mutation (K670N/M671L) (APP_770_-Swe) or empty vector (-) control. Cell harvest and western blotting were performed as described in Section 2. Antibodies 6E10 and Ab5 were used as controls for APP and GAPDH as a loading control. The mobility of molecular mass markers is indicated on the left side of each immunoblot.

We generated a novel mouse monoclonal antibody 3C11 specific to Aβ by immunizing mice with a chimeric protein composed of 21–140 α-synuclein followed by Aβ 1–42. This method takes advantage of several properties of α-synuclein, such as its high solubility and immunogenicity that allow it to act as a carrier protein. Initial ELISA screening using the immunogen as well as Aβ 42 and α-synuclein determined that 3C11 is specific for Aβ 42. Using ELISA method 1 (see Section 2), further characterization using synthetic peptides spanning the Aβ 42 molecule (Table 1) demonstrates that the epitope for 3C11 requires amino acids before 11 since 3C11 did not react with a Aβ 11–42 peptide, but bound to Aβ 1–16, 1–28, and 1–42 peptides (Figure 1A). Ab5 is a previously characterized monoclonal antibody with a determined epitope within Aβ 1–16 and served as a control [55]. Similar to 3C11, Ab5 was immunoreactive for Aβ 1–16, 1–28, and 1–42 peptides and was also negative for Aβ 11–42 (Figure 1B). ELISA characterization using additional synthetic peptides toward the N-terminal region of Aβ was used to further characterize the antigenic determinant of the novel monoclonal antibody 3C11. 3C11 was reactive for Aβ 1–16, Aβ 1–20, and Aβ 1–40 peptides with some slight immunoreactivity for Aβ 2–14 (Figure 1C,E). Since 3C11 did not react with peptide Aβ 4–16, its epitope must require amino acids before Phe4. Since 3C11 did not detect Aβ 1–12, its epitope must also require residues in the stretch between amino acids His13-Lys16 (Figure 1C,E). Ab5 was immunoreactive for peptides Aβ 2–14, 1–16, 1–20, and 1–40, but not Aβ 6–18, Aβ 8–20 or Aβ 4–16 (Figure 1D,F). Ab5 was only modestly reactive with Aβ 1–12 by ELISA (Figure 1D,F). From these results, it can be surmised that the epitope for Ab5 extends from Aβ residues 2 or 3 and Aβ residues 13 and 14 are needed for optimal binding. Immunoblotting analysis using overexpressed amyloid precursor protein (APP) isoform 770 in HEK293T cells shows that 3C11 demonstrates strong specificity for APP, which was also detected by Aβ monoclonal antibodies 6E10 and Ab5 (Figure 2).

### 3.2. Immunohistochemical Staining of Aβ Pathology Using Novel Monoclonal Antibody 3C11

**Figure 3:**
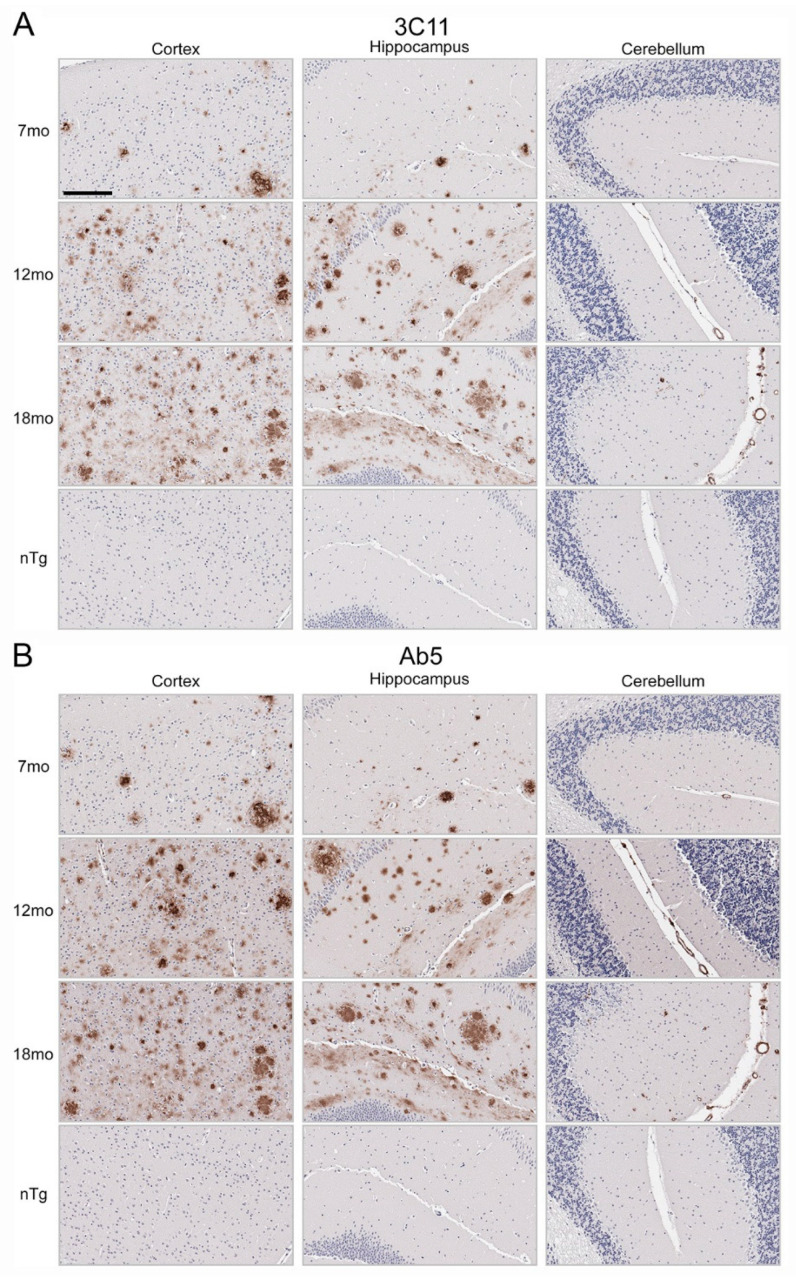
Immunohistochemical staining of Aβ deposits in brain tissue of TgCRND8 mouse model of amyloidosis. Immunohistochemical staining using monoclonal antibodies 3C11 (**A**) and Ab5 (**B**) of 7-, 12-, and 18-month-old TgCRND8 and 7-month-old nTg mice as described in Section 2. The scale bar represents 150 µm.

**Figure 4:**
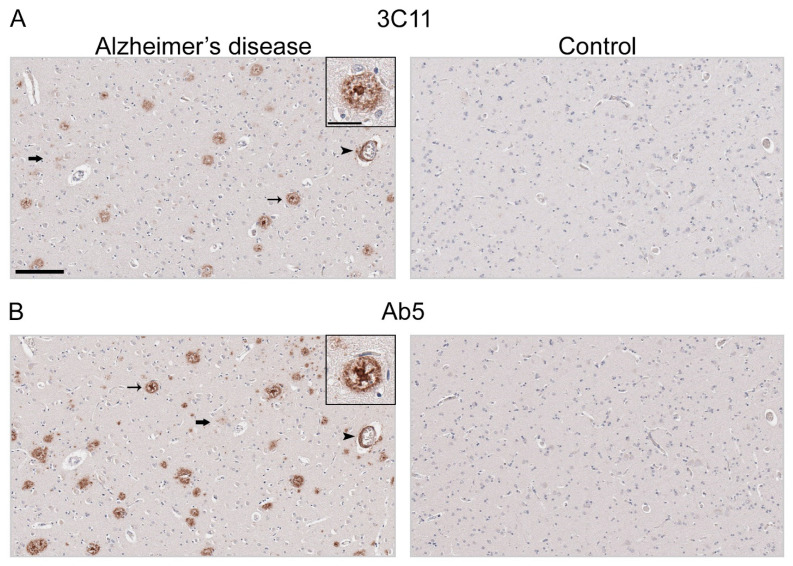
Immunohistochemical staining of Alzheimer’s disease human brain tissue using antibody 3C11. Immunohistochemical staining of AD and control mid-frontal cortical tissue using antibodies 3C11 (**A**) and Ab5 (**B**) was performed as described in Section 2. Immunohistochemical staining of cases AD-1 and Control-1 are shown. Demographics of cases are shown in Table 2. Thin arrows indicate 3C11 or Ab5 immunoreactive core plaques, thick arrows indicate diffuse plaques, and arrowheads indicate cerebral amyloid angiopathy. Scale bars of low magnification images represent 150 µm, and the inset is 35 µm.

**Figure 5:**
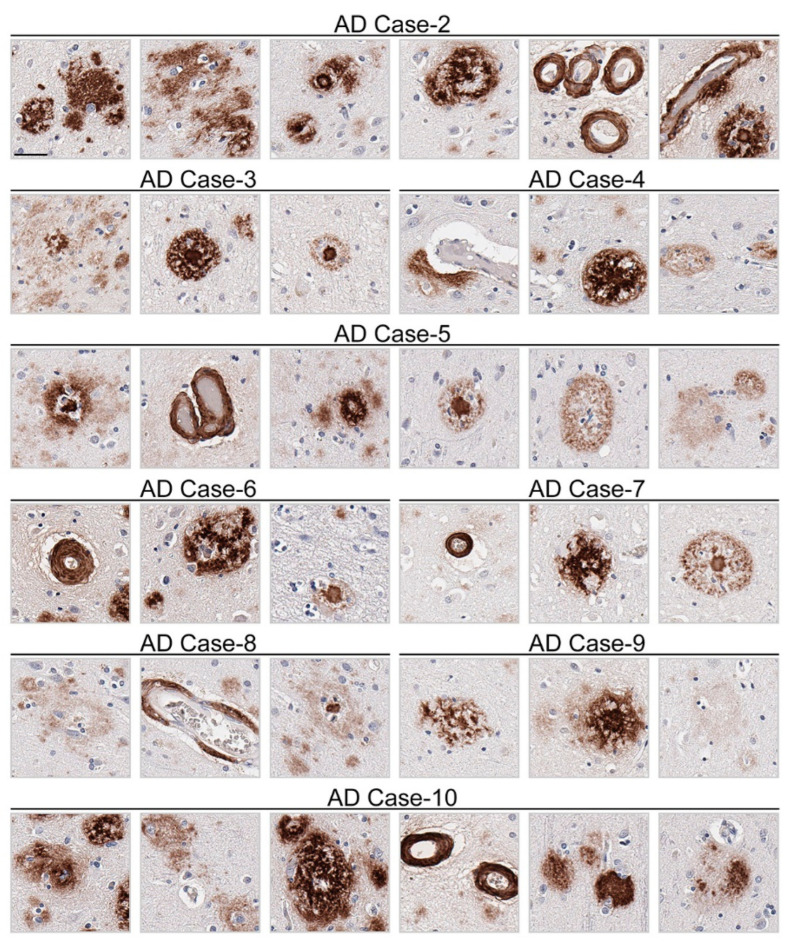
Appreciable diversity of Aβ pathology detected by monoclonal antibody 3C11. Immunohistochemical staining of Aβ pathology using monoclonal antibody 3C11 in Alzheimer’s disease cases is shown. Demographics of cases are shown in Table 2. The scale bar represents 35 µm.

Immunohistochemical staining using 3C11 of brain tissue from the TgCRND8 mouse model of amyloidosis [58] demonstrates immunoreactivity of Aβ plaque pathology at 7-, 12-, and 18-month time points (Figure 3A). The cortex, hippocampus, and cerebellum were investigated, as they are regions affected by Aβ plaque deposition during the progression of AD [2]. Both the cortex and hippocampus demonstrated a temporal increase of Aβ plaque pathology detected by 3C11 (Figure 3A). Both 3C11 immunoreactive cored and diffuse plaques were detected throughout the neuropil, and CAA was observed in the cerebellum, which was more apparent at 12 and 18 months (Figure 3A). Ab5 demonstrated a similar pattern of immunoreactivity of Aβ plaques and CAA pathology as 3C11 (Figure 3B). We did not observe immunocytochemical staining in non-transgenic (nTg) mouse tissue using 3C11 or Ab5 (Figure 3A,B). Next, we performed immunohistochemical staining of post-mortem human brain tissue from high AD and control cases, where we examined the mid-frontal cortex, a region affected by Aβ deposition in AD [2]. Immunostaining with 3C11 revealed robust immunoreactivity of Aβ plaques within the parenchyma as well as CAA within cortical blood vessels (Figure 4A). Similar results were obtained with Ab5 (Figure 4B). We next quantified 3C11 and Ab5 immunoreactive dense Aβ plaques within the frontal cortex of a subset of high AD cases. We found that 3C11 was generally less sensitive when comparing the ratio of dense plaques stained with each antibody (mean = 0.81, standard deviation = 0.26). However, depending on the AD case, 3C11 immunoreactivity overall mirrored or was substantially less than that of Ab5 (Table 3). A vast assortment of morphological subtypes of Aβ deposits were observed in the AD cases immunohistochemically stained using 3C11 herein (Figure 5). CAA within the vessel walls of arterioles and arteries and perivascular Aβ pathology were detected (Figure 5). Aβ plaque morphology detected using 3C11 was also appreciably diverse; we observed several types of Aβ plaques throughout the parenchyma using 3C11 (Figure 5). 3C11 stained diffuse plaques, which exhibited typical characteristics: they were widespread, ranged in size, and had ill-defined borders. 3C11 immunoreactive classic cored-plaques were detected, where both the core and corona could be distinguished (Figure 5).

## 4. Discussion

In this study, we present the characterization of a novel mouse monoclonal antibody specific for Aβ. Monoclonal antibody 3C11 was found to have an epitope within the N-terminal region of Aβ determined by ELISA, react with APP overexpressed in cultured cells, and immunohistochemically stain parenchymal Aβ plaque and cerebrovascular pathology in the TgCRND8 mouse model of amyloidosis and post-mortem human brain tissue from individuals neuropathologically diagnosed with AD.

Aβ is a product of the metabolic processing of the type 1 transmembrane protein APP by proteases within the membrane, resulting in several fragments [61,62,63,64]. Following successive cleavage by endoproteases, generated Aβ peptides can be found coalesced into fibrils deposited in blood vessels and as plaques in the extracellular space in AD [7,64]. The region in APP where Aβ is found is targeted by both α and β secretases toward the N-terminal region, which is followed by the release of several truncated products [64]. The antibody described herein was raised against a chimeric protein of αS 21–140 fused to Aβ 42. Using synthetic peptides spanning the Aβ 42 molecule, we determined that the epitope for the novel antibody was located within the N-terminal region. Similar to the carboxy-terminus of Aβ, the amino-terminal region has been shown to affect aggregation and is also subject to post-translational modifications [65]. Truncation within the amino-terminal region of Aβ has been shown to affect fibrillization kinetics determined by in vitro aggregation assays [66,67], suggesting the N-terminus plays a critical role in modulating the folding of Aβ peptides into a conformation suitable for polymer formation. In characterizing the epitope of 3C11, we utilized synthetic peptides devoid of any post-translational modifications that are found in human AD brain tissue. Based on our ELISA data (Figure 1), we found that the 3C11 epitope is within the N-terminal half region as it binds similarly to Aβ 1–16 compared to Aβ 1–40 (Figure 1C,E). Since it did not recognize Aβ 4–16 it requires residues before Phe4. Furthermore, since it did not recognize Aβ 4–16 the epitope must require residues His13-Lys16; however, we cannot conclude that this is a linear epitope. Post-translational modifications that occur within the N-terminal region of Aβ, such as phosphorylated Ser8 or pyroglutamylation at residue 3, which reside within the Aβ molecule [65], could affect 3C11 immunoreactivity; this topic was not investigated herein. We did find that APP expressed in cultured cells, a system in which post-translational modifications can occur, was immunoreactive for 3C11, suggesting the 3C11 epitope on APP is present under native conditions. Additionally, several autosomal dominant AD mutations also lie within the N-terminal region of Aβ, which have been shown to have remarkably different effects on fibril morphology and aggregation rates [66,68].

Aβ lesions are characterized by their histological properties, revealed through the use of several dyes and immunocytochemistry methods [5]. These methods reveal the scope of morphologically diverse deposits throughout the brain, while notably, immunohistochemistry is able to be used to detect Aβ species in both amyloid and non-amyloid states, which can often be missed by other classical methods that are biased toward the detection of protein conformation rather than the protein itself. Our novel antibody 3C11 successfully reacted with Aβ deposits in the TgCRND8 mouse model of amyloidosis at several time points. In the TgCRND8 mouse tissue, 3C11 immunolabelling extended throughout the cortex and hippocampus to include the staining of diffuse and cored plaques, both of which were quite abundant, as previously described [58]. Remarkably, 3C11 was also found to immunohistochemically stain Aβ neuropathology in cortical tissue from patients with a primary neuropathological diagnosis of high AD. 3C11 immuno-labeled aggregated Aβ within the blood vessel walls throughout the frontal cortex in several AD cases with CAA. Utilizing Ab5 as a comparison antibody for Aβ neuropathology, we determined the relative immunoreactivity of 3C11 and Ab5 by counting dense Aβ plaques in a subset of AD cases. We found that the sensitivity of 3C11 for dense plaques was overall lower compared to that of Ab5 (Table 3), although we only quantified dense plaques immunostained by 3C11 in high AD cases. Nevertheless, we observed that 3C11 bound to plaques of several morphologies, including diffuse and cored plaques throughout the neuropil, highlighting its utility in detecting several notable neuropathological hallmarks of AD.

The addition of 3C11 to the repertoire of Aβ monoclonal antibodies will be beneficial for future studies in which the utility of passive immunotherapy using different antibodies can be investigated. The novel Aβ antibody described herein will aid in the investigation of both APP and its proteolytically derived cleavage product, Aβ, in models of altered proteostasis to further elucidate mechanisms underlying AD pathogenesis. Additionally, 3C11 will be valuable for immunohistochemical studies investigating Aβ plaque deposition in AD and other neurodegenerative diseases.

## Figures and Tables

**Table 1:** Synthetic Aβ peptides used in this study.

Peptide	Primary Amino Acid Sequence
Aβ 1–12	DAEFRHDSGYEV
Aβ 2–14	AEFRHDSGYEVHH
Aβ 4–16	FRHDSGYEVHHQK
Aβ 1–16	DAEFRHDSGYEVHHQK
Aβ 6–18	HDSGYEVHHQKLV
Aβ 8–20	SGYEVHHQKLVFF
Aβ 1–20	DAEFRHDSGYEVHHQKLVFF
Aβ 1–28	DAEFRHDSGYEVHHQKLVFFAEDVGSNK
Aβ 17–24	LVFFAEDV
Aβ 23–42	DVGSNKGAIIGLMVGGVVIA
Aβ 11-42	EVHHQKLVFFAEDVGSNKGAIIGLMVGGVVIA
Aβ 1–40	DAEFRHDSGYEVHHQKLVFFAEDVGSNKGAIIGLMVGGVV
Aβ 1–42	DAEFRHDSGYEVHHQKLVFFAEDVGSNKGAIIGLMVGGVVIA

**Table 2:** Summary of cases used in this study.

Cases	Primary Neuropathological Diagnosis	Secondary Neuropathological Diagnosis	Tertiary Neuropathological Diagnosis	Thal Phase	Braak Stage	CERAD	APOE	Sex	Age
AD-1	AD high	CAA widespread, mild to moderate	LATE stage 2	4	VI	Frequent	3/4	f	82
AD-2	AD high	CAA widespread, moderate		5	VI	Frequent	4/4	m	70
AD-3	AD high	CAA widespread, moderate	LBD amygdala predominant	5	V	Frequent	3/3	f	82
AD-4	AD high	CAA widespread, moderate		5	VI	Frequent	4/4	m	72
AD-5	AD high	CAA, moderate, widespread		5	V	Frequent	3/4	f	86
AD-6	AD high	CAA widespread, moderate	LATE NC stage 2, hippocampal sclerosis	4	V	Frequent	3/4	f	87
AD-7	AD high	CAA widespread, moderate		5	V	Frequent	3/3	f	78
AD-8	AD high	CAA widespread, moderate	LBD limbic-transitional	5	VI	Frequent	3/3	f	81
AD-9	AD high	CAA widespread, moderate	LATE stage 1	5	V	Frequent	4/4	f	71
AD-10	AD high	CAA widespread, moderate		5	VI	Frequent	3/4	m	63
AD-11	AD high	CAA focal, mild		5	V	Frequent	3/4	f	85
AD-12	AD high	CAA widespread, moderate to severe		3	V	Frequent	4/4	f	82
AD-13	AD high	LBD diffuse neocortical		5	VI	Frequent	3/4	f	84
AD-14	AD high	CAA widespread, moderate to severe	cortical microinfarcts	5	V	Frequent	3/4	f	85
AD-15	AD high	CAA widespread, moderate to severe		5	V	Frequent	3/4	m	80
AD-16	AD high	CAA widespread, mild to moderate		5	V	Frequent	3/3	f	83
AD-17	AD high			5	VI	Frequent	3/3	f	76
AD-18	AD high	CAA widespread, moderate	LATE NC stage 2, hippocampal sclerosis	5	VI	Frequent	3/4	f	99
AD-19	AD high	CAA (moderate)	LATE NC 1	5	VI	Frequent	3/4	m	81
AD-20	AD high	CAA widespread, mild	LBD limbic-transitional	5	VI	Frequent	4/4	f	76
AD-21	AD high	LBD amygdala predominant	CAA focal, mild	5	VI	Frequent	4/4	f	75
AD-22	AD high	LBD amygdala predominant	CAA focal, mild	5	VI	Frequent	3/3	m	70
AD-23	AD high	CAA widespread, mild to moderate	LBD amygdala predominant	5	VI	Frequent	3/3	m	78
AD-24	AD high	LBD amygdala predominant		5	V	Frequent	3/3	f	88
Control-1	PART, definite, Braak II			0	II	None	3/3	f	72
Control-2	No significant pathological findings			0	0	None	3/4	f	55
Control-3	PART, definite, Braak I			0	I	None	2/3	f	73

AD Alzheimer’s disease, APOE apolipoprotein E, CAA cerebral amyloid angiopathy, CERAD Consortium to Establish a Registry for Alzheimer’s disease, LATE-NC limbic-predominant age-related TDP-43 encephalopathy neuropathological change, LBD Lewy body disease, PART primary age-related tauopathy. Primary neuropathological diagnosis of AD was determined using established criteria and guidelines [59,60].

**Table 3:** Quantification of 3C11 immunoreactivity in AD human brain tissue.

Case	3C11/Ab5 Immunoreactive Positivity of Dense Aβ Plaques
AD-1	0.55
AD-2	0.92
AD-4	0.52
AD-7	0.90
AD-8	0.86
AD-10	0.85
AD-11	0.98
AD-20	0.60
AD-21	1.32
AD-22	1.10
AD-23	0.60
AD-24	0.51
N	12
Mean of all cases (Standard deviation)	0.81 ± 0.26

## Data Availability

The datasets used and analyzed from the current study are available from the corresponding author upon reasonable request.
